# Pain among nursing home patients in the Netherlands: prevalence, course, clinical correlates, recognition and analgesic treatment – an observational cohort study

**DOI:** 10.1186/1471-2318-7-3

**Published:** 2007-02-14

**Authors:** Martin Smalbrugge, Lineke K Jongenelis, Anne Margriet Pot, Aartjan TF Beekman, Jan A Eefsting

**Affiliations:** 1 Department of Nursing Home Medicine, VU University Medical Center, Amsterdam, The Netherlands; 2 Institute of Extramural Medicine (EMGO), VU University Medical Center, Amsterdam, The Netherlands; 3 Trimbos-institute, Netherlands Institute of Mental Health and Addiction, Utrecht, The Netherlands; 4 Department of Psychiatry, VU University Medical Center, Amsterdam, The Netherlands

## Abstract

**Background:**

Pain is highly prevalent in nursing homes (NH) in several countries. Data about pain in Dutch NH's, where medical care is delivered by specifically trained NH-physicians, are not available. The aim of the present study is to determine prevalence, course, correlates, recognition and treatment of pain among Dutch NH-patients and to make a comparison with international data.

**Methods:**

The study-population consisted of 350 elderly NH-patients from 14 Dutch NH's. Pain (pain-subscale Nottingham Health Profile) and clinical characteristics (gender, age, cognition, depression, anxiety, sleeping problems, morbidity and functional status) were measured at baseline and at six months.

Association of pain (baseline and six months) with clinical characteristics was assessed with chi-square and multiple logistic regression analyses.

**Results:**

Pain-prevalence was 68.0% (40.5% mild pain symptoms, 27.5% serious pain symptoms). 80% of the patients with pain at baseline still experienced pain at six months. Serious pain at baseline was significantly associated with depression (OR: 2.56; 95% CI: 1.34-4.89) and anxiety (OR 2.47; 95% CI: 1.22-4.99). Serious pain at six months was associated with pain at baseline (OR 18.55; 95% CI: 5.19-66.31) and depression at baseline (OR: 2.63; 95% CI:1.10-6.29). Recognition of pain by NH-physicians varied (35% to 69.7%) depending on measurement instrument and severity of pain. Analgesics were received by 64.5% (paracetamol (acetaminophen), NSAIDs, opioids). Paracetamol (acetaminophen) and opioids frequently were prescribed below daily defined doses.

**Conclusion:**

Pain occurred frequently also among Dutch NH-patients and was associated with depression and anxiety. Recognition and treatment by NH-physicians proved sub-optimal. Future studies should focus on interventions to improve recognition and treatment of pain.

## Background

One of the main goals of nursing home care is preservation of the best possible quality of life. A very important aspect of quality of life is being free of pain. Prompt recognition and adequate treatment is therefore requested when nursing home patients are suffering from pain.

Previous studies in several countries showed that pain is a common problem in the long-term care setting [1-3]. Prevalence rates ranged from 27% to 83%, with the highest rates in studies that used patients self-report. One longitudinal study furthermore indicated that pain is chronic in many patients [4]. Factors found to be associated with pain included depression, anxiety, impaired sleep, comorbidity, reduced mobility and decreased involvement in recreational activities [5-7]. Recognition of pain by nursing home staff is poor and frequently no treatment is given [3].

For the Netherlands, in which nursing home physicians are employed by nursing homes for medical care and in which nursing home medicine is an independent medical specialism with its own specific training programme [8,9], epidemiological data about pain in nursing homes are lacking until now. More knowledge about pain in Dutch nursing homes can contribute to a further improvement of the quality of medical care and the quality of life of its residents.

The aim of the present study was therefore (a) to determine prevalence, course, correlates, recognition and treatment of pain in Dutch nursing home patients and (b) to make a comparison with international data.

## Methods

### Study population

This study is based on data collected in the Amsterdam Groningen Elderly Depression (AGED) study [10]. Fourteen nursing homes in the North West of the Netherlands were selected to participate. Nursing homes for specific disease categories were excluded as were small nursing homes (<60 beds). No large reorganization or rebuilding activities were allowed because of possible influence on the mood of the respondents. To be eligible, subjects had to be aged 55 years and over, speakers of Dutch and able to communicate sufficiently, without serious hearing problems or severe cognitive impairment (Mini-Mental State Examination >= 15) [11].

Patients with at baseline an expected stay of less than 6 months were excluded. All eligible patients were informed verbally and in writing. Written informed consent was obtained from all respondents prior to inclusion. The study received approval from the Medical Ethics Committee of the VU University Medical Center.

Between November 1999 and May 2001, data were collected. All measurements were administered twice in a face to face interview: at baseline and six months later. Sampling procedures are described in detail elsewhere [10].

From the source population (696 nursing home patients who met inclusion criteria) eventually an active sample of 350 patients remained who participated in the baseline data-collection. 58 patients (8.3%) died before the interview could be started and 46 patients (6.6%) could not be interviewed because they were suffering from acute illness, terminal illness or coma. 235 patients (33.8%) refused to participate in this study and 7 patients (1.0%) were not included for other reasons.

In the second wave at six months 229 (65.4% of the original 350) patients participated; 43 (12.3%) patients died; 16 (4.6%) patients had severe cognitive impairment; 9 (2.6%) patients were unable to communicate; 15 (4.3%) patients were hospitalised or moved; 35 (10%) patients refused and 3 (0.9%) patients were lost to follow-up for other reasons.

### Measurement instruments

#### Measurement of pain

Perceived pain was measured with the pain subscale of the Dutch version of the Nottingham Health Profile, which measures pain by selfreport [12]. The pain subscale of the Nottingham Health Profile contains 8 items with a yes-no format and a score ranging from 0 to 8 (0 = no pain symptoms). Cronbach's alpha was 0.70 in the present study. The internal consistency of the reliability of the pain scale in previous studies [12-14] proved also to be satisfying (Cronbach's alpha:0.70–0.85) as was the test-retest reliability (Spearman's r: 0.84).

The items of the pain subscale concern pain intensity as well as situations in which pain occurs (i.e. 'suffering unbearable pain' or ' pain when walking': see table 1).

Pain was therefore used in analyses as an ordinal variable and not as a continuous variable. Painstatus was scored as 0 = no pain, as 1 = mild pain symptoms or as 2 = serious pain symptoms. Patients with serious pain symptoms had to report 'unbearable pain' or 'constant pain'. Patients with mild pain symptoms reported positive on other items but had no 'unbearable pain' and no 'constant pain'.

Recognition of pain was measured by asking the attending nursing home physician at baseline if the patient experienced pain in the past two weeks. Medical care in Dutch nursing homes is delivered on a daily basis by specially trained and registered nursing home physicians. Recognition was also measured by recording if patients were treated with analgesics. Analgesic drug use, including the prescribed daily dose/defined daily dose ratio (PDD/DDD-ratio), was assessed by chart review and was classified according to the Anatomical Therapeutic Chemical (ATC) classification system [15]. The PDD/DDD-ratio is used as an indication of the adequacy of dosing. Three main groups of analgesics were distinguished: paracetamol (acetaminophen), non-steroidal anti-inflammatory drugs (NSAIDs) and opioids.

Prescription of antidepressants and of anxiolytics/hypnosedatives (mainly bezodiazepines) was also registrated.

#### Measurement of correlates

Demographic characteristics like age and gender were gathered by chart review. Cognitive functioning was assessed with the Mini-Mental State Examination (MMSE) [11]. Sum scores were dichotomized: a score between 15 and 23 referred to the presence of cognitive impairment. A cut-of score of 15 was chosen as exclusion criterion: required for a reliable use of the Geriatric Depression scale.

Depressive symptoms were measured with the Geriatric Depression Scale [16]. A score of >=11 was considered to be indicative for the presence of clinically relevant depressive symptoms.

Anxiety was measured with the Schedules for Clinical Assessment in Neuropsychiatry [17]. Anxiety symptoms were defined as presence of phobic complaints or presence of feelings of anxiety or panic in the last four weeks.

Sleep was also measured with the SCAN. Presence of impaired sleep was defined as having troubles falling asleep or being awake in the night (both for at least one hour, three or more times a week, during at least one month) or waking up early (two hours earlier than normally, three or more times a week, during at least one month).

Information about the presence of physical illnesses was obtained from the attending physician using a questionnaire containing thirteen main groups of somatic diseases. The total number of physical illnesses was dichotomized on the median.

Data on functional limitations were supplied by care personnel involved in direct daily care, using the Groningen Activity Restriction Scale (GARS): 11 items concerning Activities of Daily Living [18]. Sum scores (11–44) were dichotomized on the median. Higher scores mean more disability.

### Statistical analyses

Analysis of attrition was done by comparing baseline characteristics (chi-square analyses) of patients who could be interviewed at baseline and at six months with baseline characteristics of patients who could only be interviewed at baseline.

Prevalence of pain was calculated at baseline and at six months. The relations between pain at baseline and demographic and clinical characteristics were evaluated by calculating crude odds ratios (OR) and corresponding 95% confidence intervals (CI). As a next step multiple logistic regression was used to calculate adjusted odds ratios. Only variables with a statistically significant (p < 0.05) crude odds ratio were entered in the multiple logistic regression model.

To identify baseline characteristics that were associated with presence of pain at six months the same analyses were done with pain at six months as dependent variable. Presence of pain at baseline was entered in these analyses also as an independent variable.

The course of pain was described in two ways. Firstly, at NHP symptom level. For all eight NPH items the course of the symptom over six months was assessed (see table 1). Secondly, the course was described for the constructed ordinal pain variable (no pain, mild pain symptoms, serious pain symptoms).

Prevalence of analgesic use and prescription of antidepressants and of anxiolytics/hypnosedatives were calculated at baseline and at six months and differences in use in patients with and without symptoms of pain were assessed.

## Results

### Sample and attrition

Demographic and clinical characteristics are shown in table 2. About two thirds of the sample were women, their mean age was 79.3 (SD 8.3) years. All patients had moderate to severe functional impairments.

Analysis of attrition showed no statistically significant differences in baseline characteristics between patients who participated in data-collection at baseline and at six months, and patients who only participated at baseline.

### Prevalence, risk-indicators and course

The prevalence of pain was 68.0% at baseline: 40.5% (n = 138) had mild pain symptoms and 27.5% (n = 94) had serious pain symptoms.

Table 3 shows that presence of serious pain symptoms at baseline was statistically significant associated in multiple logistic regression analysis with presence of depressive symptoms and presence of anxiety symptoms. This association was not found for presence of mild pain symptoms.

Presence of serious pain symptoms at six months was in multiple logistic regression analysis statistically significant associated with presence of pain (OR 18.55; 95% CI 5.19–66.31) and depressive symptoms (OR 2.63; 95% CI 1.10–6.29) at baseline. Presence of mild pain symptoms at six months was only statistically significant associated with presence of pain at baseline (OR 3.83; 95% CI 1.94–7.55).

Data about the course of pain over six months were available for 216 patients. Especially 'unbearable pain' (58.6%) and 'constant pain' (66.0%) are persistent over time (table 1). Nearly 80% (121/153) of the patients with pain at baseline still reported pain after six months (table 4).

### Recognition and treatment

Presence of mild pain symptoms was recognized in 34.4% and presence of serious pain symptoms was recognized in 38.2% of the patients, when measured by interviewing the attending physician.

Pain-recognition was also measured by assessing prescription of analgesics (see table 5). Pain was then recognized in 61.2% of the patients with mild pain symptoms and in 69.7% of the patients with serious pain symptoms.

Table 5 shows that treatment with analgesics occurred frequently in the present population: 54.5% (158/290) had analgesics prescribed at baseline, prescription as needed included (8.6%). Analgesic prescription rate was the highest among patients who reported pain (64.5%, pnr included). Paracetamol (acetaminophen) was the most frequently prescribed analgesic, followed by NSAIDs and opioids.

The observed differences in analgesic therapy (monotherapy, combination therapy, prescription as needed, no therapy) between patients with pain (mild pain symptoms and serious pain symptoms) and without pain were statistically significant (Pearson chi-square 34.26; df 6; p < 0.0001). Further analyses showed no significant difference in analgesic therapy between patients with mild pain symptoms and serious pain symptoms (Pearson chi-square 3.44; df 3; p = 0.30).

The majority of analgesic users at baseline still reported pain. Only 31 (19.6%) of them were free of pain, 74 (46.8%) still experienced mild pain symptoms and 53 (33.5%) still experienced serious pain symptoms.

Data of analgesic use at six months depicted similar results (data not shown).

Considering the adequacy of dosing, opioid-presciptions had in 12.5% a PDD/DDD-ratio of 1 and in 69.2% a PDD/DDD-ratio lower than 2/3. Paracetamol (acetaminophen) prescriptions showed in 15.5% a PDD/DDD ratio of 1 and in 30.8% a PDD/DDD-ratio lower than 2/3. NSAIDs were prescribed in 84.0% with a PDD/DDD-ratio of >= 1 and in 16.0% with a PDD/DDD-ratio below 1.

Combinations of analgesics were prescribed to 12.1–15.8% of the patients with pain at baseline.

There was no significant difference in antidepressants prescription (Pearson chi-square 2.42; df 2; p = 0.30) between patients with pain (mild pain symptoms and serious pain symptoms) and without pain, but patients with pain (mild pain symptoms and serious pain symptoms) used significantly more frequently anxiolytics/hypnosedatives than patients without pain (Pearson chi-square 6.39; df 2; p = 0.04). Further analyses showed no significant difference in prescription of anxiolytics/hypnosedatives between patients with mild pain symptoms and patients with serious pain symptoms (Pearson chi-square 0.64; df 1; p = 0.42).

## Discussion

### Prevalence, risk-indicators, course

The prevalence of self-reported pain was high in this population of nursing home patients: 40.5% of the patients reported mild pain symptoms and an additional 27.5% serious pain symptoms at baseline. Only 32.0% was free of pain. This was in line with previous studies which used self-reported pain as method of pain measurement [5,19-22]. Pain further appeared to be persistent: nearly 80% of the patients with pain at baseline still reported pain after six months. A rather alarming observation was that 'unbearable pain' and 'constant pain', the most serious NHP-pain items, also tended to be persistent (58.6% and 66.0%) over a six months period.

Important associated characteristics with serious pain symptoms at baseline were presence of depressive symptoms and presence of anxiety symptoms. Serious pain symptoms at six months were associated with pain at baseline and with presence of depressive symptoms.

The observed association with depressive symptoms and anxiety symptoms was also found in previous investigations [5]. In a longitudinal study the association between pain and depressive symptoms was found to be intimate and reciprocal [23]. As a consequence, when assessing and treating pain, attention should also be paid to diagnosing and treating depressive and anxiety symptoms.

Contrary to previous studies, no association of self-reported pain with cognitive functioning was observed [20,24]. This could be caused by the fact that in the present study only patients with a MMSE-score >=15 were included, which implies that more severely demented patients were excluded.

### Recognition and treatment

Recognition of pain measured by interview and recognition of pain measured by prescription of analgesics resulted in different recognition rates: 35–40% vs. 61.2–69.7%, presumably because they reflect two ways of defining pain recognition. Analgesics prescription as measurement for pain recognition indicates that at some moment (before or after admission to the nursing home) a physician diagnosed pain and prescribed an analgesic.

A positive interview-answer as measurement for pain recognition indicates that the physician recognized that the patient experienced pain at the time of the interview.

Both methods of measuring pain recognition made clear that presence of pain in many patients remained undetected. Previous studies showed similar results, not only for recognition of pain by physicians but also for recognition by nursing staff [25].

False attitudes about pain and age among elderly patients and their caregivers and non-communication about pain between patients and caregivers were held responsible for this underdetection [25].

Treatment with analgesics occurred frequently in the present population: 54.5%. Analgesic prescription rate was the highest among patients who reported pain (64.5%, pnr included). Paracetamol (acetaminophen) was used the most frequently, followed by NSAIDs and opioids. In spite of use of analgesics many patients still reported pain. Underdosing of analgesics and a restrained prescription of combinations of analgesics and of opioids could be potential causes.

Underdosing, for which a PDD/DDD-ratio below 1 is indicative, was indeed observed, especially for opioids (PDD/DDD-ratio < 2/3: 69.2%) and to a lesser extent also for paracetamol (acetaminophen) (PDD/DDD-ratio < 2/3: 30.8%). PDD/DDD-ratio for NSAIDs on the other hand was relatively high (84% PDD/DDD-ratio >=1) in view of the risk of renal failure and hypertension for older patients [26]. A similar pattern of underdosing was found in a pharmaco-epidemiological study in six nursing homes [27].

Combinations of analgesics were prescribed to 12.1–15.8% of the patients with pain at baseline. Given the fact that these patients still had pain, one would expect a higher rate of combinations of analgesics.

Opioids were prescribed to 11.7% of the patients with pain at baseline and about half of the opioid-prescriptions were given as monotherapy. This seems not in line with pain-guidelines which advise opioids to be added to NSAIDs and paracetamol (acetaminophen) as a next step in analgesic treatment [28].

The high prevalence of pain in the present study and the observations made about recognition and treatment of pain suggest that in nursing homes in the Netherlands, pain-management is suboptimal just as was found in studies among nursing home populations in other countries, despite the deliverance of medical care by specifically trained nursing home physicians.

Assessment of pain by a standardized instrument at regular times, which is uncommon in the Netherlands (use of screening instruments like the Resident Assessment Instrument [29] is not mandatory) and interdisciplinary discussion of the assessment results by nursing staff and attending physician, may help to improve pain-management in nursing home patients [30]. The interdisciplinary discussion also offers a good opportunity for better implementation of recently published pain-guidelines [28].

Future studies should investigate if a standardized pain assessment and an interdisciplinary discussion of the assessment have a positive effect on pain among nursing home patients.

### Limitations

The present study has some limitations, which warrant comment. Firstly, the study-population is a selective one. Serious cognitive impairment (MMSE <15), speech and language problems, severe physical illness and expected discharge within six months were important exclusion criteria.

Secondly, attrition was considerable as could be expected among the frail population of elderly patients residing in nursing homes. Nevertheless, analysis of attrition showed no differences in distribution of baseline-characteristics between patients who remained in the study and patients who dropped out.

Thirdly, pain was measured with the NHP-pain subscale which contains items concerning different aspects of pain (pain-intensity as well as items concerning situations in which pain is present). We therefore constructed an ordinal pain variable, that in our opinion has satisfying clinical face validity by using intensity and long lasting presence of pain for defining more severe pain.

## Conclusion

Pain is a common and usually persistent problem among nursing home patients.

Important associations are observed with depressive and anxiety symptoms.

Recognition by nursing home physicians in the Netherlands is comparable to recognition in other countries and open for improvement.

Despite a high prescription rate of analgesics, many patients still report pain. This may be caused by underdosing and restrained use of analgesic combination therapy.

To warrant optimal quality of life of nursing home patients improvement of pain recognition and treatment is needed. Introduction of standardized pain assessments at regular times, treatment according to pain guidelines and interdisciplinary discussion of the assessment results and treatment effects, may help to establish this.

## Competing interests

The author(s) declare that they have no competing interests.

## Authors' contributions

All authors contributed to the study conception and design. MS participated in the data analysis and drafting of the manuscript, LJ participated in the data collection, AP participated in the data analysis, AB participated in the data analysis, JE participated in the data analysis. All authors critically reviewed the manuscript, read and approved the final manuscript.

## Pre-publication history

The pre-publication history for this paper can be accessed here:



## Figures and Tables

**Table 1:** Pain symptoms measured by the pain section of the Nottingham Health Profile (NHP) in a sample (n = 350)^a ^of Dutch nursing home patients at baseline and after six months.

	*baseline n (%)*	*six months n (%)*
**Nightly pain**	75 (34.7%)	68 (31.5%)
		(38 (50.7%) persistent)^b^
**Unbearable pain**	29 (13.4%)	34 (15.7%)
		(17 (58.6%) persistent)
**Pain when changing position**	88 (40.7%)	83 (38.4%)
		(57 (64.8%) persistent)
**Pain when walking**	45 (20.8%)	44 (20.4%)
		(23 (51.1%) persistent)
**Pain when standing**	49 (22.7%)	41 (19.0%)
		(17 (34.7%) persistent)
**Constant pain**	50 (23.1%)	47 (21.8%)
		(33 (66.0%) persistent)
**Pain when stair climbing**	7 (3.2%)	3 (1.4%)
		(1 (14.3%) persistent)
**Pain when sitting**	89 (41.2%)	66 (30.6%)
		(44 (49.4%) persistent)

^a ^Data pain section NHP at baseline and after six months available for 216 patients.

^b ^The number (%) of patients in whom after six months the symptom still is present

**Table 2:** Demographic and clinical characteristics of a sample of nursing homepatients in the Netherlands (n = 350).

Characteristic	N	%
Age (mean 79.3; SD 8.3; range:55–99)		
55–79	169	48.3
80–99	181	51.7
Sex		
Male	109	31.1
Female	241	68.9
Length of stay		
> 1 year	209	59.7
< 1 year	141	40.3
Cognitive functioning (MMSE) (mean 22.0; SD 3.8; range 15–30)		
15–23	221	63.1
24–30	129	36.9
Depressive symptoms (GDS >= 11)		
Present	155	44.3
not present	195	55.7
Anxiety symptoms (n = 333)		
Present	99	29.7
not present	234	70.3
Impaired sleep (n = 326)		
Present	89	27.3
not present	237	72.7
Number of physical illnesses (n = 300) (mean 3.7; SD 1.6; range 1–9)		
<=3	145	48.3
>3	155	51.7
Functional impairments (n = 340)		
Severe	172	50.6
Moderate	168	49.4

**Table 3:** Clinical characteristics associated with presence of pain at baseline in a sample (n = 350)^a ^of Dutch nursing home patients.

*Characteristic*	Mild pain symptoms n = 138		Serious pain symptoms n = 94	
	Crude OR (95% CI)^b^	Adjusted OR (95% CI)^c^	Crude OR (95% CI)^b^	Adjusted OR (95% CI)^c^
	
Age (>=80/<80)	0.83 (0.50–1.37)		0.61 (0.35–1.07)	
Sexe (female/male)	1.11 (0.65–1.88)		1.48 (0.81–2.70)	
MMSE-score (>23/<=23)	1.13 (0.67–1.91)		1.33 (0.75–2.34)	
Depressive symptoms (yes/no)	1.67 (0.98–2.84)		**3.55 (1.98–6.35)**	**2.56 (1.34–4.89)**
Anxiety symptoms (yes/no)	1.82 (0.97–3.38)		**3.77 (1.98–7.17)**	**2.47 (1.22–4.99)**
Sleeping problems (yes/no)	1.76 (0.94–3.29)		**2.55 (1.32–4.90)**	1.87 (0.92–3.79)
Number of physical illnesses (>3/<=3)	1.23 (0.72–2.11)		1.66 (0.91–3.03)	
Functional impairments (large/moderate)	0.95 (0.58–1.58)		1.34 (0.77–2.34)	

^a ^Complete data of all variables were available for 293 patients

^b ^Crude odds ratio (OR) and 95% confidence intervals (95% CI). Controlgroup (n = 109): patients without pain.

^c ^Adjusted odds ratio derived from a multiple logistic regression model. As independent variables were entered variables with a statistically significant crude OR. Controlgroup (n = 109): patients without pain.

^d ^Statistically significant OR (p < 0.05) in bold characters.

**Table 4:** Course of pain symptoms in a sample (n = 350)^a ^of Dutch nursing home patients over six months.

	***Follow-up (6 months****)*			
	No pain	Mild pain symptoms	Serious pain symptoms	**Total**

***Baseline***				
**No pain**	39	21	3	**63**
**Mild pain symptoms**	27	52	16	**95**
**Serious pain symptoms**	5	14	39	**58**
**Total**	**71**	**87**	**58**	**216**

^a ^Data about pain symptoms at baseline and after six months available for 216 patients.

**Table 5:** Pain and prescription of analgesics, antidepressiva and anxiolytica/hypnosedativa at baseline in a sample (n = 350)^a ^of Dutch nursing home patients.

	No pain n = 93	Mild pain symptoms n = 121	Serious pain symptoms n = 76
***Analgesic monotherapy***	25 (26.9%)	53 (43.8%)	33 (43.4%)
Paracetamol^b^	14 (15.1%)	31 (25.6%)	13 (17.1%)
NSAIDs	7 (7.5%)	20 (16.5%)	11 (14.5%)
Opioids	4 (4.3%)	2 (1.7%)	8 (10.5%)
Other analgesics	0 (0.0%)	0 (0.0%)	1 (1.3%)
***Analgesic combination therapy***	0 (0.0%)	10 (12.1%)	12 (15.8%)
Paracetamol and NSAIDs	0 (0.0%)	5 (4.1%)	4 (5.3%)
Paracetamol and opioids	0 (0.0%)	5 (4.1%)	5 (6.6%)
Paracetamol, NSAIDs and opioids	0 (0.0%)	0 (0.0%)	2 (2.6%)
Opioids and opioids	0 (0.0%)	0 (0.0%)	1 (1.3%)
***Analgesic prn***^**c**^	6 (6.5%)	11 (9.1%)	8 (10.5%)
***No analgesic***	62 (66.7%)	47 (38.8%)	23 (30.3%)
***Antidepressiva***	20 (21.5%)	30 (24.8%)	26 (34.2%)
***Anxiolytica/hypnosedativa***	34 (36.6%)	66 (54.5%)	37 (48.7%)

^a ^Complete pain data and prescription data at baseline were available of 290 patients.

^b ^Paracetamol = acetaminophen

^c ^Prescription as needed: did not occur with standard prescription of analgesics in this sample.
